# Evidence of repeated zoonotic pathogen spillover events at ecological boundaries

**DOI:** 10.3389/fpubh.2024.1435233

**Published:** 2024-11-05

**Authors:** Antoine Filion, Mekala Sundaram, John Paul Schmidt, John M. Drake, Patrick R. Stephens

**Affiliations:** ^1^Department of Integrative Biology, Oklahoma State University, Stillwater, OK, United States; ^2^Department of Infectious Diseases, University of Georgia, Athens, GA, United States; ^3^Savannah River Ecology Laboratory, University of Georgia, Aiken, SC, United States; ^4^Odum School of Ecology and Center for the Ecology of Infectious Diseases, University of Georgia, Athens, GA, United States

**Keywords:** disease ecology, Schmalhausen’s law, ecological boundaries, spillover, filovirus, disease emergence

## Abstract

Anthropogenic modifications to the landscape have altered several ecological processes worldwide, creating new ecological boundaries at the human/wildlife interface. Outbreaks of zoonotic pathogens often occur at these ecological boundaries, but the mechanisms behind new emergences remain drastically understudied. Here, we test for the influence of two types of ecosystem boundaries on spillover risk: (1) biotic transition zones such as species range edges and transitions between ecoregions and (2) land use transition zones where wild landscapes occur in close proximity to heavily impacted areas of high human population density. Using ebolavirus as a model system and an ensemble machine learning modeling framework, we investigated the role of likely reservoir (bats) and accidental host (primates) range edges and patterns of land use (defined using SEDAC categories) on past spillover events. Our results show that overlapping species range edges and heightened habitat diversity increase ebolavirus outbreaks risk. Moreover, we show that gradual transition zones, represent by high proportion of rangelands, acts as a buffer to reduces outbreak risks. With increasing landscape changes worldwide, we provide novel ecological and evolutionary insights into our understanding of zoonotic pathogen emergence and highlight the risk of aggressively developing ecological boundaries.

## Introduction

The past few decades have been marked by a drastic increase in zoonotic pathogen spillover events worldwide (1–3). The recent COVID-19 pandemic was an unfortunate demonstration of the dire effects that zoonotic pathogen spillover can have on human and animal health (4). In the face of the rising number of emerging zoonotic pathogens (1), the One Health approach, which considers human, animal and environmental health simultaneously, has been proposed as a powerful conceptual tool to help tackle the rise of emerging zoonotic pathogens (5). However, a major component leading to pathogen spillover events, environmental health, is often neglected and stands as a deficiency to providing a truly comprehensive One Health approach (6). To prepare for and mitigate future zoonotic pathogen emergence, a better understanding of how environmental factors influence disease risk is needed. Ecosystem boundaries have been suggested as a common environmental factor that may contribute to zoonotic spillover by being areas where many host species can come into contact, fostering cross species transmission and potential spread of pathogens into human populations (7). However, direct empirical tests of whether ecosystem boundary areas have higher spillover risk than other regions remain surprisingly rare. Further, even in cases where this hypothesis has been tested [e.g., (8, 66)] it remains unclear whether ecological and evolutionary factors, the simple confluence of human and wild animal populations, or both contribute to spillover risk.

Broadly speaking, ecosystem boundaries can be thought of as reflecting two types of transitions zones (1) biotic transition zones between ecoregions or biomes, which can also manifest as areas where the edges of many species geographic ranges occur, and (2) land use transition zones, where transitions from wild to anthropogenically dominated landscapes occur, and thus where human populations are likely to come into contact with a wide variety of potential disease reservoir host species. Whether these two types of transition zones influence spillover risk, and if so which does so most strongly, has infrequently been tested empirically (23). This is important because the mechanisms by which biotic vs. land use transition zones are likely to influence spillover risk, and whether they would even necessarily be expected to do so, somewhat differ.

If we define a biotic transition zone as an area where many species ranges edges occur, two macroecological hypotheses have important implications for expected patterns of spillover risk. The geographic center-abundant hypothesis (9) predicts that individuals of species located near their geographic range center thrive due to better overall conditions compared to those near species range edges, which results in higher reproductive success and higher abundance near the center of any given species geographic ranges (10, 11). While this theory and the validity of its predictions are still disputed (12), it focuses on the idea that the center of a species range represents an area of greater ecological suitability than range edges (13). Ecologically challenging localities at the edge of a species range could increase a reservoir’s ecophysiological stress, potentially allowing pathogens to reach higher prevalence or abundance, increasing spillover risk by increasing the chance of transmission when individuals come unto contact (7). Conversely, lower expected abundance of reservoir host species could actually decrease spillover risk by decreasing contact rates between reservoirs and other species including humans (14).

Another macroecological mechanism relating species range-edges to spillover risk is Schmalhausen’s law (15–18). Schmalhausen’s law predicts that whenever individuals in a population are pushed to the edges of their ecological or physiological tolerances, unusual phenotypes will be observed due to gene expression outside of the range of environments for which species genotypes have been canalized by previous natural selection (17, 19). In the context of spillover, this could lead to unusual population dynamics or that might affect transmission risk (15). If the range-center-abundance hypothesis is correct, species range edges represent the types of environments in which species are likely to be pushed to their physiological limits. Thus, Schmalhausen’s law can be regarded as another mechanism by which the range-center-abundance hypothesis might be related to spillover risk. So far, this theory has primarily been tested in systems involving vector-borne pathogens (15, 18, 20), though a recent study showed evidence that it may influence outbreak size in at least one directly transmitted pathogen, Marburg virus (16).

**Figure 1:**
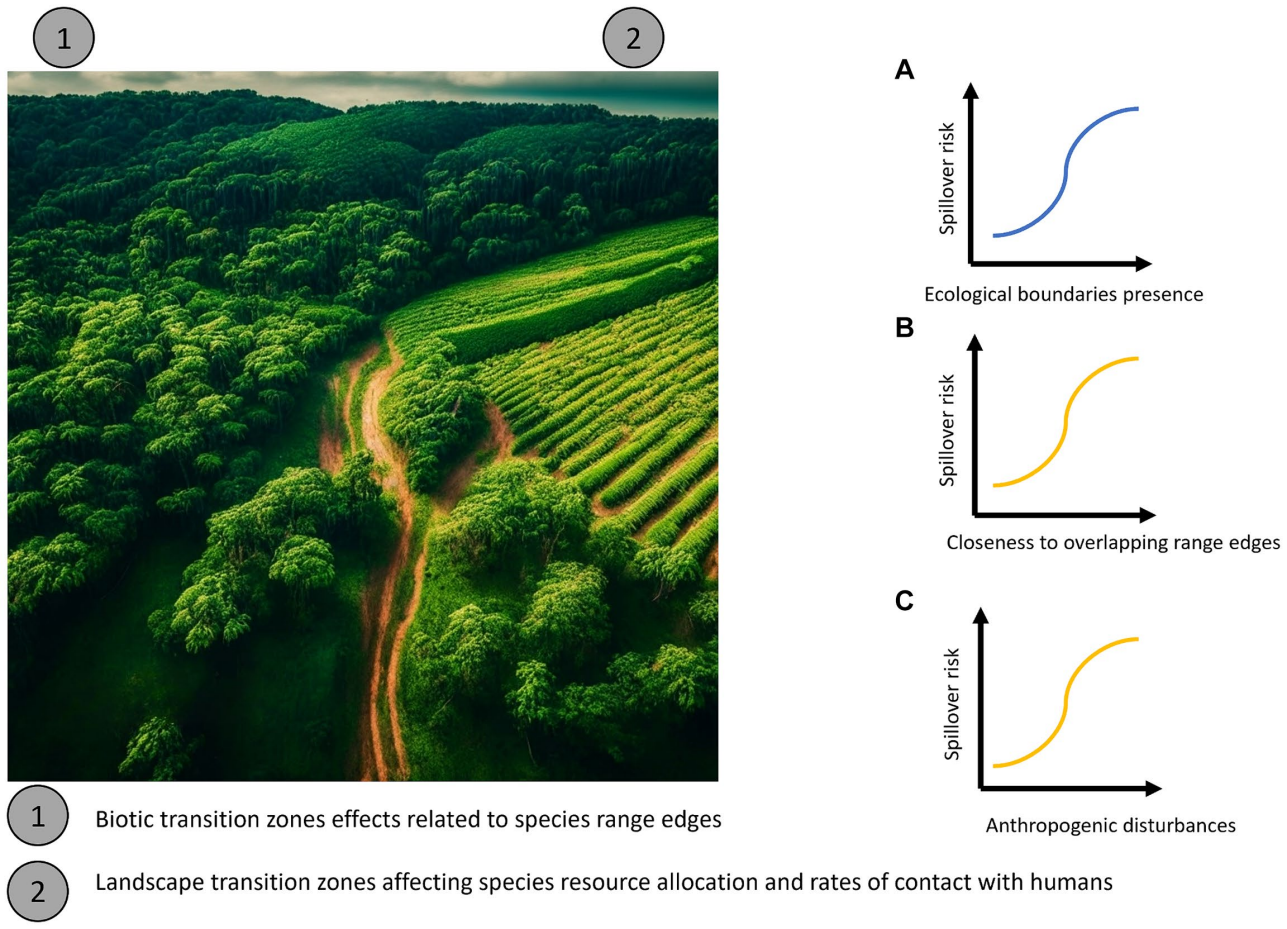
Potential main processes along with their directionality explaining the main mechanisms underlying transition zones in directly transmitted pathogens. **(A)** Expected overall directionality of spillover risk with increasing of ecological boundaries (blue). **(B)** If such patterns are generated by evolutionary mechanisms, spillover risk should be more tightly correlated with the distance to the edge of the geographic range of competent host species (orange). **(C)** If such patterns are related to artificial transition zones such as conversion to agricultural landscapes or unnatural ecotones, spillover risk should be more tightly correlated with the proportion of land being used for croplands or rangelands that mark the transition between wild and settled areas (orange).

In contrast to purely biotic transition zones, we define land use transition zones as areas where wild habitats, relatively unaffected by anthropogenic change and with low human population density, transition into urban or agricultural landscapes with much higher human population density (Figure 1). These unnatural ecological boundaries, forming transition zones between environments (21), are hypothesized to be fertile grounds for the emergence of zoonotic pathogens (2). For instance, the extinction-filter evolutionary hypothesis predicts that species that evolve in high-disturbance areas should be more resilient to anthropogenic environmental changes, such as human encroachment and habitat fragmentation (22). Thus, in disturbed habitats these species will be among the survivors and be able to reach high abundance. If such a species is a disease reservoir, it could greatly increase spillover risk in disturbed habitats (23). Conversely, even if species that evolved in stable environments are able to survive in these areas, they will presumably still be under great stress (22), which could also lead to higher pathogen prevalence and increased spillover risk. More, simply land use transition zones might represent “mixing zones” between wild and settled areas, where human populations from highly impacted settled areas tend to come into contact with populations of host species being maintained in less impacted wild areas, such as forests, and to which these transitional zones are still “permeable” (7).

Few studies have considered both types of transition zones simultaneously (in fact none that we are aware of), and thus tested which sets of mechanisms are most likely to influence spillover risk. However, if biotic transitions zones are more important to driving spillover, we would expect spillover risk to increase near areas where many species geographic ranges occur (Figures 1A,B). Conversely, if land use transition zones are more important (Figure 1C) we would expect spillover to occur in regions where both human-impacted and relatively undisturbed wild areas occur in close proximity. While land use transition zones will certainly represent new range edges for some species that cannot tolerate close association with humans in settled areas, in general anthropogenic disturbances in the environment are expected to affect the population dynamics, and thus range boundaries, of different species idiosyncratically (24). Co-occurrence of multiple species range-edges would only be expected to strongly predict spillover risk if biotic transitions zones *per se* are affecting zoonotic disease dynamics. The confluence of both types of transition zones might also lead to even higher spillover risk than either alone.

Regardless, to establish which, if any, of the mechanisms we discuss may be at play in driving spillover risk, a first step is to ask whether biotic transition zones, land use transition zones, or both show increased spillover risk. Ebolaviruses [by which we here refer to any African species of *Orthoebolavirus sensu* (25)] present a compelling study system for this exercise. Ebolavirus outbreaks produce extremely high mortality in human populations, often in excess of 50% (26). Likely because of this, ebolavirus is the subject of intense monitoring and research effort (27), and we speculate that a larger proportion of spillover events have likely been documented compared to many other sporadically occurring pathogens in tropical regions. Ebola virus is also a generalist pathogen that can infect a wide variety of species including bats, ungulates, and primates including humans (26), and thus a pathogen in which biotic transition zones (i.e., areas where many host species geographic range boundaries overlap) might be expected to influence spillover risk. Although fruit bats have been strongly implicated as a reservoir (28), the source of spillover events into human populations has often been primates (29). Collectively, the spatial ranges of the bat and primate species that are susceptible to ebolaviruses cover most of sub-Saharan Africa and all of the areas where ebolavirus spillover events have occurred. It is thus possible to combine large scale land-use and species geographic range data in an integrative framework to test whether species range edges or patterns of land use most strongly affect spillover risk (Figure 1).

While a number of studies have investigated the drivers of Ebolavirus spillover risk [e.g., (8, 30–32)], few studies have specifically tested for species range edge-effects or contrasted their influence with that of transition zones related to patterns of human land use. Here, we investigate the role of natural biotic and anthropogenically created ecological boundaries in contributing to repeated disease spillover events using the Ebola virus as a model system. We quantify biotic transition zones based on the overlapping range edges of the most likely primary (bats) and secondary amplifying (primates) hosts (28). We define land use transitions zones as areas where relatively pristine and human-altered land use types occur in close proximity. Using this system, we make two predictions: (1) Due to their high potential for ecological disturbances, land-use transition zones will be important in contributing to higher outbreak risks. (2) If Schmalhausen’s principles hold true, areas closer to biotic transition zones of Ebola virus reservoirs or accidental hosts range edges will have a higher outbreak risks. Using ensemble machine-learning methods and a presence pseudo-absence framework, we test whether models of biotic transition zones, land use transitions zones, or that include both best predict the location of sites where previous Ebola virus spillover events have been documented.

## Methods

### Outbreak data collection

We compiled all reported Ebola outbreaks with human cases from Kuhn (26), the Centers for Disease Control (33), and ProMED (34). We then pruned the dataset to include only outbreaks confirmed to be Ebola virus (including both species *Sudan ebolavirus* and *Zaire ebolavirus*) by laboratory testing and that originated from a wild source (e.g., we excluded data points from accidental laboratory spillover events). For each outbreak, we compiled the date of outbreak, its geographical origin, and the number of human cases, allowing us to work with 44 outbreaks ranging from 1976 to 2020, inclusively (and excluding outbreaks reported after we initiated our work).

### Pseudo-absence matrix collection

Following previous studies of filoviruses and other pathogens [e.g., (16, 32, 35)], we investigated the spatial characteristics of localities where outbreaks originated using a presence/pseudo-absence approach where the sites at which outbreaks are known to have originated are compared to randomly selected localities in the same regions (e.g., background points). First, we converted a shapefile of Africa to raster [package: Raster (36)] with a 50 km grid square. Then, we defined strict land boundaries based on regions where the environmental conditions are broadly similar to those where outbreaks have occurred in the past (32), and performed a seeded random sampling to create our pseudo-absence dataframe (R code is provided in the Supplementary material).

### Anthropogenic biomes data collection

To quantify patterns of land use we used the Anthropogenic Biomes of the World V1 raster (37) from the Socioeconomic Data and Application Center (SEDAC) to assign one of six large categories of land use to each locality in Africa: (1) dense settlements, (2) villages, (3) croplands, (4) rangelands, (5) forested, (6) wildlands. We used version 1, downloaded March 17th 2023 at a gridded resolution of 0.083 km2, for analyses.

### Species ranges

We obtained the spatial ranges of all fruit bat (Pteripodidae) and primate species in sub-Saharan Africa as a polygon shapefile from the IUCN red list repository (38). We focused on these species because Sundaram et al. (28) showed that they are likely to be the most important primary and secondary amplifying hosts (respectively) and because firsthand accounts of past outbreaks highlight their importance (26, 39). For each presence and pseudo-absence locality we extracted the minimum distance to the edge of the nearest geographic range polygon for each species using the dist2line function [package: geosphere (40)], representing a minimum distance between an Ebola virus outbreak and the distributional edge of either natural reservoirs of Ebola virus or accidental hosts [packages: sp. (41); Raster]. We then calculated the median distance between an outbreak event and the range-edges of all bat species and all primate species, separately, as the variables to use in our models, representing the areas where many species range-edges overlap, representing biotic transition zones.

### Species edge composition

We extracted the spatial edges of all our bat and primate species by converting each species polygon file into a spatial line object (package: sp), and using the dist2line function (package: geosphere) to create a 40 km buffer around the geographic range edge of each species. We chose a 40 km buffer because preliminary analyses showed that nearly all Ebola virus outbreaks happen within 40 km of the range-edges of the suspected reservoirs of Ebola virus (see Supplementary Figure S1). We then matched buffer zones with the anthropogenic biomes data to capture land use composition at the edge of our species ranges.

### Landscape composition around outbreaks locations

We calculated the same 40 km buffer around our outbreak and pseudo-absence locations, and then extracted all geographic coordinates in that buffer. We then matched these coordinates with the anthropogenic biomes data (37) to capture land use composition near outbreaks locations. We next calculated the percentage area of each of the six types of land use tracked by the SEDAC anthropogenic biomes data within each zone. To determine whether habitat conversion to a specific landscape type or highly fragmented habitat drive Ebola virus spillover risk, we calculated a Simpson diversity index [package: vegan (42)] for the anthropogenic biomes data around the outbreaks and pseudo-absence locations. *A priori*, we expected that areas with high values of Simpson’s diversity index (SDI) would be more likely to contain areas with both relatively pristine and human-altered land use types in close proximity.

However, high values of SDI alone do not necessarily indicate that human-altered and wild land use types occur in the same general area, it only indicates multiple land use types are present and no one type dominates in terms of relative area. To determine whether high values of SDI were associated with areas that could often be considered land use transition zones (in the sense of both wild and human-dominated landscapes occurring together), we divided land use types into human-dominated (agriculture, villages, and urban areas) and wilderness/low human-population densities (wildlands, rangelands, and forest). We then determined whether the 40 km radius around each presence and pseudoabsence locality contained only human-dominated land use types, only wilderness land use types, or at least one example of both so that we could test whether areas that contained examples of both exhibited significantly higher SDI values.

### Data analysis

All analyses were performed using R version 4.0.2 (43). We provide R code as Supplementary material to allow readers to fully reproduce our results and have access to our models’ conditions. Prior to modeling, all our predictor variables were checked for collinearity. We then removed redundant predictor variables to reduce model variance, following the recommendations of Elith et al. (44) for boosted regression trees analyses with small data sets. We focused on gradient-boosted regression trees (GBM) for our models, implemented in caret v. 6.0–86 (45). This ensemble machine learning method only requires the user to specify distribution of the response variable (i.e., the correct link function), and is robust to the inclusion of predictor variables with virtually any underlying distribution and with complex patterns of covariation, including spatial correlation (44). We resampled the rows of the full database prior to the analysis to avoid data clumping (i.e., all outbreaks and pseudo-absences together). To avoid artificially inflating the AUC scores of models, we used down sampling to avoid overrepresenting pseudo-absence points. We checked all variable importance using Final Model call (45). Variables that had a zero status were considered as having little importance as predictors. Furthermore, all variables that had relative influence scores of less than 10% were considered non-important, somewhat arbitrarily, and we did not investigate their directionality using marginal plots (see below) or include them in subsequent models. For all models, we used (1) presence (1) or absence (0) of a documented Ebola virus outbreak at each locality as the response, with a Bernouilli distribution as the link function in our boosted regression tree analyses; (2) 2/3 of our database to train the model and 1/3 as holdout data; and (3) 10-fold cross-validation with 100 replicate to validate their robustness and to ensure that models were not overfit.

We constructed several sets of models, in each case using presence vs. background as the response variable with down-sampling of background localities to match the number of presence localities. To assess the importance of ecological vs. anthropogenic environmental factors we compared the AUC scores of models based on species geographic range edges to models based on patterns of land use (Figure 1).

To investigate the effects of host range edges on outbreak risk, we first constructed a model based on the median of the distance to the range edge of all bat or primate species, contrasting the relative influence of bat vs. primate range edges. To further disentangle the potential role of these two highly correlated predictors (R^2^ = 0.89), we built a separate model for each of them individually, and compared their AUC scores. Second, to understand the importance of bat and primate species identity in contributing to Ebola virus outbreak risk we constructed a model where we compared the relative influence of proximity to the range-edge of all bat and primate species that overlapped with the origin of at least one outbreak. As there was high potential of correlation among the many predictors, we replicated this model 99 times to minimize background noise using the “replicate” function in R.

To investigate the role of anthropogenic landscape structuring in contributing to Ebola virus outbreak risk, we used the percentage of coverage of each of six types of land type (e.g., Dense settlements, Villages, Croplands, Rangelands, Forested, Wildlands) and Simpson’s diversity index based on the number and relative area of these land use types within the 40 km buffer zone around outbreak and pseudoabsence localities as predictors.

To see whether models that included both land use and biotic transition zones better predicted spillover risk than models that included only one of these factors, we included all the variables with relative influence >10% in previous models for a final combined analysis. In this model, we used the median distance to bat species range edge, the median distance to primate species range edge, and the landscape characteristics surrounding our mapped data points (rangelands and SDI).

## Results

Overall, our database consisted of 44 known outbreak locations and 440 pseudo-absence data points. All confirmed outbreaks overlapped with at least one bat species range (see Supplementary Figure S2). Both bats and primate range edges were dominated by agricultural land uses (both forest and cropland), reaching as high as 93% of overall edge composition for one bat species (*Casinycteris argynnis*) and > 95% for a few primate species (see Supplementary Figure S3 for further details).

### Distance to the median of species edges

We identified a negative influence of both the median distance to the range edge of any species of fruit bat and the median distance to any primate range edge, with spillover risk decreasing with increasing distance to both bat and primate range edges (AUC = 0.80, Table 1A). Overall, the model including only median distance to bat range edge performed better (AUC = 0.79) than the one including only median distance to primate range edge (AUC = 0.76).

**Table 1:** Results of all boosted regression tree models.

A. Results for the evolutionary model			
Parameter	Relative influence score	Directionality	Model AUC
Median distance to bat species range edge	70.68	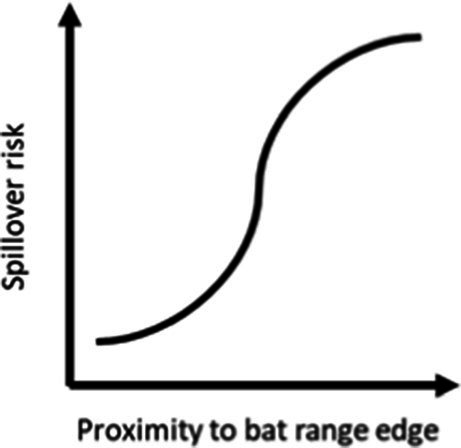	0.796
Median distance primate species range edge	29.32	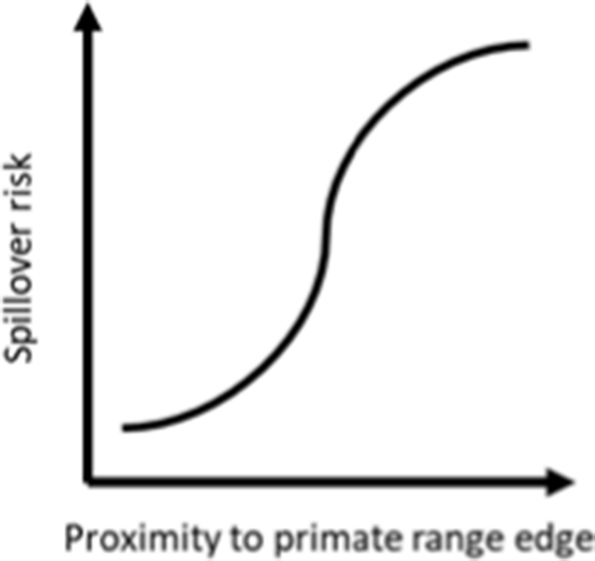	

B. Results for the landscape model			
Parameter	Relative influence score	Directionality	Model AUC
Rangelands frequency	58.75	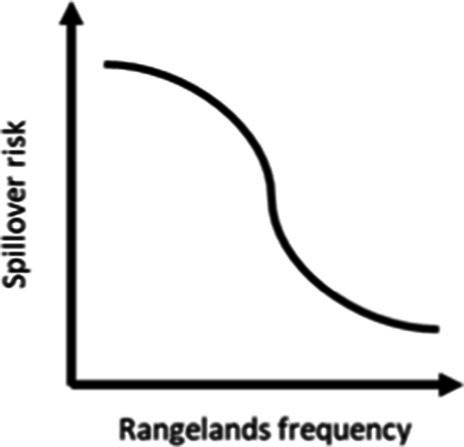	0.885
Simpson diversity index	28.03	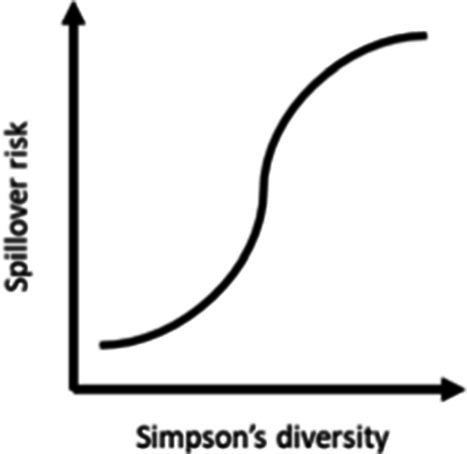	
Agricultural forest frequency	4.70	Not shown	
Wildlands frequency	3.22	Not shown	
Croplands frequency	2.95	Not shown	
Villages frequency	2.31	Not shown	
Dense settlements frequency	0	Not shown	

C. Results for the combined model			
Parameter	Relative influence score	Directionality	Model AUC
Rangelands frequency	41.00	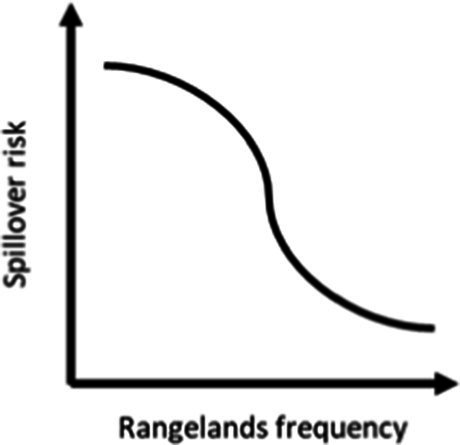	0.893
Median distance to bat species range edge	29.86	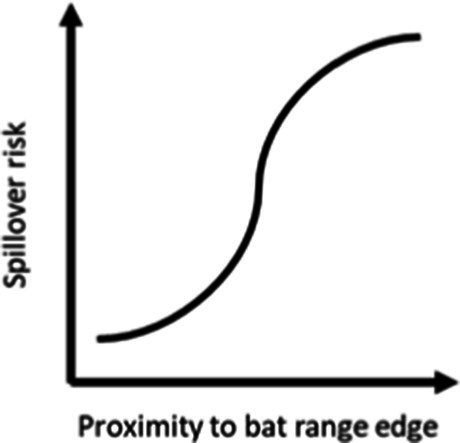	
Simpson diversity index	25.65	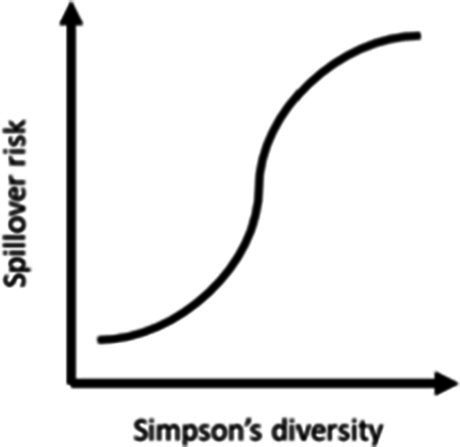	
Median distance to primate species range edge	3.45	Not shown	

Sum of relative influence scores for each model is equal to 1. Models with high AUC values have a higher probability of correctly predicting Ebola spillover risk. Directionality represents the overall effect based on marginal plots (shown in Supplementary material).

### Species importance in contributing to spillover events

**Figure 2:**
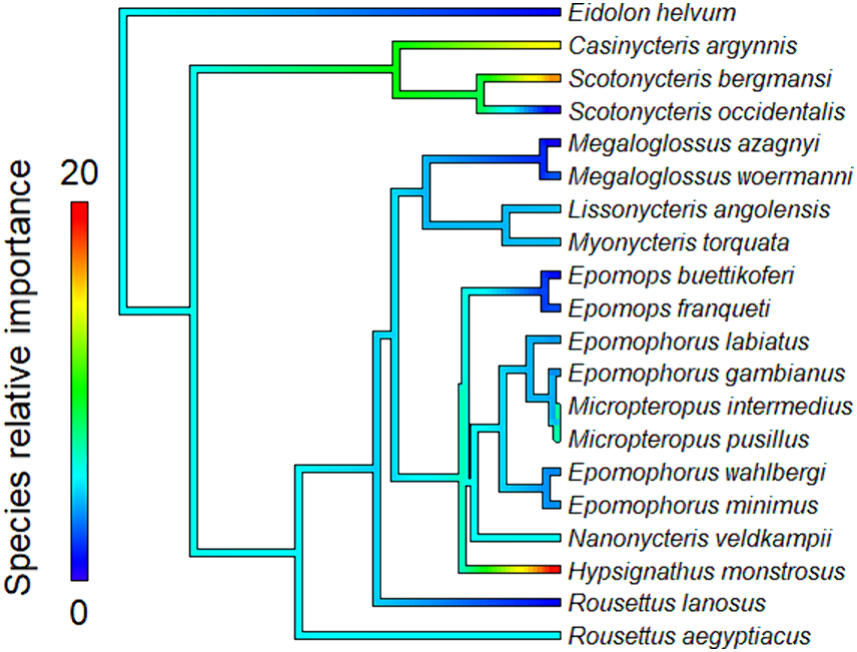
Relative influence scores of bats species in models of Ebola spillover risk.

**Figure 3:**
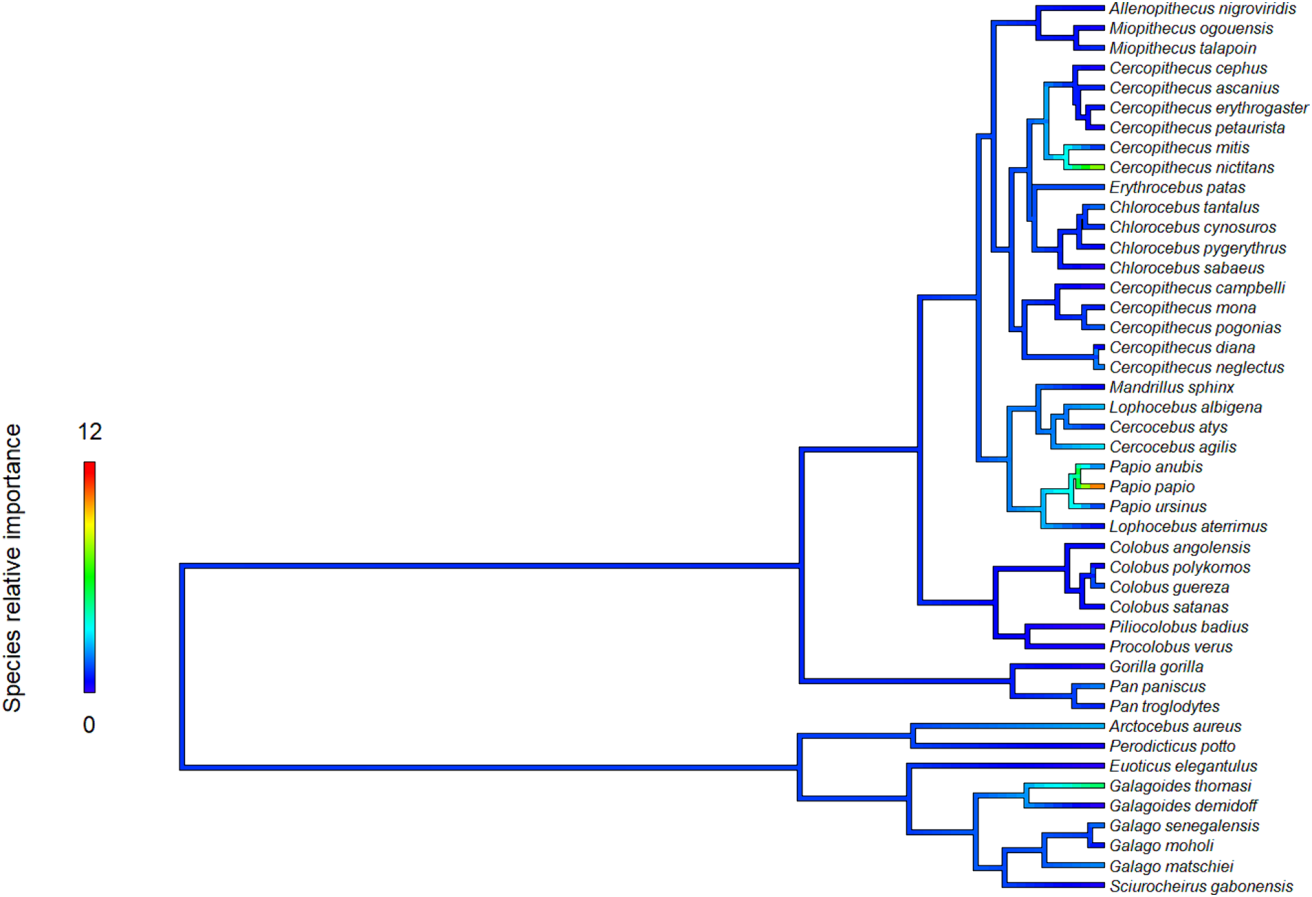
Relative influence scores of primates species in models of Ebola spillover risk.

The proximity to range edge of only a handful of species showed high relative influence scores in models of Ebola virus outbreak risk. Among bats, *Scotonycteris bergmansi*, *Hypsignathus monstrosus*, and *Casinycteris argynnis* all had high (>10%) relative influence scores (see Figure 2). Among primates, only *Papio papio* had a high relative influence (see Figure 3).

### Landscape composition around outbreaks

We identified two main landscape effects driving Ebola virus spillover risk. The relative proportion of Rangelands had the highest relative influence score in this model, negatively affecting Ebola virus outbreak risks (Table 1B), with areas with higher frequency of rangelands showing less outbreak risk. The next most influential variable was Simpson diversity index for land use types, with high values associated with higher Ebola virus spillover risk (AUC = 0.88, Table 1B). No other type of landscape had an influence score over 10%. *Post hoc* analyses also confirmed that SDI was significantly higher in areas that had at least one wilderness and one human-dominated land use type (see Supplementary Table S1).

### Combined model

Among the predictors that had a at least a 10% relative influence score in the previous models, we show that three predictors, namely proportion of rangelands, median distances to bat range edges and Simpson diversity index are the most influential drivers of Ebola spillover risk (AUC = 0.89, Table 1C). The only other predictor included in this model, median distance to primate range edge, had weak or no effect.

## Discussion

Our results suggest that both biotic and land use transition zones are associated with heightened risk of Ebola virus spillover into human populations. Specifically, areas where the median distance to the range-edges of bat species are low increased Ebola virus spillover risk (Table 1A). Moreover, we demonstrated that high SDI values, related to conversion of wild landscapes, where interactions between human and wildlife are limited, into high heterogeneity landscapes increases spillover risk. Rangelands was the land use type with the highest relative influence scores in both our Landscape and Combined models (Tables 1B,C).

### Biotic transition zones outbreak pattern

Ebola virus outbreak risk is associated with proximity to overlapping range edges of both potential reservoirs (fruit bats) and secondary amplifying hosts (primates, Table 1A). Marginal plots also showed a clear increase in outbreak risk closer to overlapping range-edges (Supplementary Figure S4). While the processes shaping species distributional ranges are numerous, they are often associated with changes in habitat quality or overall niche suitability (46). The results we observed could be driven by either of two mechanisms. First, if the interior of species range represents areas most suitable to a species ecological niche, reduction in habitat quality near the edge of a species range could increase ecophysiological stress, limiting the resources available for allocation toward immunological defence, and potentially increasing both pathogen shedding and spillover risk (47). Second, if areas near specie range edges are near the limits of species ecological tolerances, Schmalhausen’s law would predict that species could exhibit unusual population dynamics, which in some cases could amplify rates of transmission and spillover risk (17). The relative influence score of species in models also showed clear phylogenetic trends (Figures 2, 3), indicating that variation in species traits could play an important role in determining which species influence spillover risk.

One hypothesis that concerns species trait variation and spillover risk relates to pace-of-life syndrome. The pace-of-life syndrome predicts that among-species or within-species physiological traits are optimized by natural selection along a continuum related to survival and rates of reproduction, establishing a trade-off mediated by the stability of the environment (48). Higher environmental variability at species range-edges could increase within-species trait variance through disruptive selection and require higher overall resource allocation to reproduction than to immune defences (48). To test this hypothesis, we conducted a *post hoc* analysis of the relationship between the relative influence score of species in models and two reproductive traits: (1) litter size and (2) litters per year (Figures 2, 3). However, we found no evidence of a correlation between the relative influence score of species in models and their reproductive characteristics (see Supplementary Appendix S1 for a full description of the data sources, methods and results for these analyses). Based on these preliminary analyses, there is no tendency for species with faster pace-of-life syndromes (i.e., faster reproductive rates) to have a greater influence on outbreak risk, contrary to the predictions of Merril et al. (47). Based on previous work, this is perhaps unsurprising. For example, Sundaram et al. (28) showed that, when all African mammals are considered, mammalian clades containing primary and secondary amplifying Ebola virus hosts tend to consist of species with relatively slow pace-of-life syndromes. However, this result does not rule out pace-of-life trade-offs occurring through within-species variation among individuals or populations. Phenotypic plasticity could also potentially be a major driver of outbreaks dynamics for generalist parasites (49) such as Ebola virus, which can infect a wide variety of host species (26). For example, a recent review found that trait-variance in mosquito vectors is a driver of vector-borne diseases dynamics (50). However, we are unaware of any existing data sources that would have allowed us to specifically test for the influence of within-species trait variance or phenotypic plasticity in this system. Future studies could test whether changes in niche suitability at the edge of species range are associated with increased intraspecific trait variance, and whether this facilitates pathogen shedding due to increase in stress or selection toward a faster pace-of-life.

### Landscape transition zones outbreak patterns

We show mixed evidence for the importance of anthropogenic land-use transition zones. We define these as areas where relatively undisturbed wild habitats (not exceeding a human population density of 4 person/km2 (51)) transition into anthropogenically altered and human dominated land use types. Models based on land use proportion patters had higher AUC scores than models that excluded land use patterns (Tables 1A–C). We also showed that high values of SDI were associated with increased spillover risk (Supplementary Figure S5), and areas that included both wild and human dominated land use types had significantly higher SDI values that areas that contained only one or the other (see Supplementary Table S1; Supplementary Figure S5). The factor with the highest relative influence score in models that included land use variables was also proportion of rangelands (Tables 1B,C). At first glance, this would appear to further support the land-use transition zone hypothesis.

Areas identified as rangelands by the SEDAC database are mostly comprised of lands used by humans for livestock, albeit with low population densities (37). It usually includes areas with mix of vegetations types including grasslands, shrubs and woodlands (52). Rangelands are thus unique as they cannot be classified as only one category of land use, but rather as a mix of different vegetation types and land uses (37, 51), effectively acting as a gradual transition zone between more homogenous human-dominated or wild-dominated areas. We thus consider rangelands somewhat of a “transitional” land use type in its own right. However, surprisingly, rangelands appear to reduce spillover risk while higher SDI values increases it. Marginal plot showed spillover risk was drastically reduced in areas that had more than a small percentage of rangelands (Supplementary Figure S4), and much higher in areas where it was completely absent. In fact, few of the spillover localities in our data contained any rangelands at all (Supplementary Figure S7). Given that rangelands can contain a variety of habitat types, we assumed *a priori* that rangelands might increase spillover risk by bringing species with differing microhabitat requirements into close proximity.

We are unaware of any previous studies that have directly tested for an influence of rangelands on spillover risk. However, we speculate these areas exhibit reduced spillover risk due to the either (1) low availability of habitat suitable for any individual reservoir species, which presumably only occur in relatively small and disconnected patches or (2) reduced contact rates between reservoirs and human populations. This could limit the population density of species that depend on any particular habitat, which in combination with the generally low human population density of these areas (37) could drastically lower spillover risk. Some important Ebola virus reservoirs, such as forest specialist bat species (28, 53), could also be excluded from rangelands altogether. Far from increasing spillover risk, rangelands seem to act as a buffer between human inhabited areas and areas with high reservoir abundance. While this does further support the influence of altered landscapes on spillover risk, only high SDI areas show increased risk consistent with the mechanisms proposed for land-use transition zones (2, 22, 23).

We have shown that high habitat diversity, represented by high SDI values, are correlated with both high number of different land use types and a mix of land use (see Supplementary Figures S5, S6). As such, we believe that at least two mechanisms likely contribute to heightened spillover risk in areas with high SDI. First, highly heterogenous patterns of disturbed and undisturbed landscape are known to force multiple species to interact together, increasing outbreak risks (see review by (54)). On top of that, by increasing competition for resources, these highly heterogeneous landscapes could increase stress in animals, again heightening spillover risks. Second, high disturbance rates in these areas could push species to the edge of their ecophysiological tolerance, further increasing spillover risks. Habitat fragmentation and conversion from natural habitats to agricultural lands are a major driver of disease emergence and dynamics in many disease systems (2, 55). By increasing habitat conversion and creating disturbances, anthropogenic modifications to the landscape promote the presence of generalist species, which are often pathogen reservoirs (56). The extinction-filter theory predicts that generalist species will be more tolerant to anthropogenic disturbances, and should be more resilient to anthropogenic environmental changes, therefore more abundant in disturbed habitats (22, 57). We have shown that Ebola virus spillover risk is heightened in areas with a high diversity of habitats, supporting the hypothesis that habitat generalists are important in driving spillover. Intermixed patterns of land use have also been observed to increase spillover risk across a wide range of tropical and emerging infectious disease systems (58, 59).

Perhaps our most surprising result is the finding that rangelands drastically reduce Ebola virus spillover risk. It remains to be seen whether this pattern is peculiar to Ebola viruses, many reservoirs of which are forest specialists, or whether it will apply to other zoonotic pathogens. As a potential counter example, Rift Valley Fever is spread to humans by mosquitos from ruminant reservoirs (60), species which thrive in rangelands (61). Future studies could investigate the effects of rangelands for spillover and outbreak risk in other zoonotic pathogens. Overall, our study strongly supports the influence of both biotic and land-use transition zones to driving Ebola virus spillover risk. Future work will be needed to isolate the specific mechanisms among those proposed for ecosystem boundaries and transition zones [e.g., (2, 7, 22, 23, 47)] are most influential in this system. Our study also adds additional evidence that dynamics similar to those predicted by Schmalhausen’s law (15, 17) may influence a variety of disease systems (16, 18, 20). We suggest that one particularly useful way forward would be to investigate the predictions of Schmalhausen’s law in more detail with compartmental mechanistic models [e.g., (62, 63)] where compartments near the edge of a hypothetical species range have less predictable transmission dynamics than compartments near the center. This could help refine the types of conditions under which Schmalhausen’s law like effects would be expected to contribute to increased spillover risk or larger outbreaks.

## Conclusion

Gaining a deeper understanding of the mechanisms that drives disease spillover events in transition zone is of prime importance to disease ecology. Taken together, our results highlight the importance of both biotic and land-use transition zones for predicting spillover risk. With humans encroaching further in the landscape, one could predict that humans and wildlife are bound to interact even more closely together. By changing the purpose of the landscape and increasing ecophysiological stress in already evolutionary challenging areas (e.g., biotic transition zones), there is the potential for an increase in disease outbreak risk due to unusual outbreak dynamics. With the current rate of emerging infectious zoonotic diseases potentially coming from all over the world, we bring forward a suite of mechanisms that are candidate explanations for causal processes causing severe outbreak risk in many systems. More importantly, we demonstrate that evolutionary mechanisms and anthropogenic encroachment can create synergistic effects in contributing to pathogen outbreaks, and that specific types of land conversion can affect risk in expected ways.

## Data Availability

The datasets presented in this study can be found in online repositories. The names of the repository/repositories and accession number(s) can be found at: Dryad https://datadryad.org, doi: 10.5061/dryad.zw3r228f6.
