# Supplementary material for: Evidence of repeated zoonotic pathogen spillover events at ecological boundaries
Source: Front Public Health. 2024 Nov 5;12:1435233. doi: 10.3389/fpubh.2024.1435233 (PMC11577354; doi:10.3389/fpubh.2024.1435233)
Supplement: Supplementary file 1 [file Data_Sheet_1.docx]

Supplementary material

Supplementary materials for “Evidence of repeated zoonotic pathogen spillover events at ecological boundaries”

Antoine Filion, Mekala Sundaram, John Paul Schmidt, John M. Drake and Patrick R. Stephens

**Contents:**

**Appendix S1**: Pace of life methods and results.

**Figure S1**: Miminum distance from each confirmed Ebola outbreak from any bat species range-edge.

**Figure S2:** Confirmed Ebola outbreaks with fruit bat species ranges.

**Figure S3.1:** Agricultural landscape frequency of all fruit bat species investigated in this study

**Figure S3.2:** Agricultural landscape frequency of all primate species investigated in this study.

**Figure S4:** Raw marginal plots from the GBM analysis.

**Figure S5:** Simpson diversity index for each general land use categories.

**Figure S6:** Simpson diversity index per number of land use types according to the SEDAC database.

**Figure S7:** Number of locations per proportion of Rangeland in their 40km buffer.

**Table S1:** ANOVA and Post-Hoc Tukey HSD results for Simpson’s diversity index.

**Appendix S1:** *Pace of life methods and results*

To understand whether the differences in the relative influences scores of individual bat species were a result of trait variation among species (i.e., to test the “pace-of-life” hypothesis), we downloaded the Amniote project database to incorporate reproductive life-history traits of reservoirs (bats) and accidental hosts (primates) into our database (Myhrvold et al. 2015), namely litter size (the number of young in a single litter) and number of litters per year. We then used a Bayesian model (package: BRMS; Burkner et al. 2017) with weakly informative priors (provided in the R code supplemental file), to test the influence of the two traits mentioned above on the calculated relative influence score separately.

Overall, species traits were poor predictors of species relative influence scores (Litter size effect size: bats = 6.32 (Lower CI= -1.66, Upper CI=37.85); primates = -0.22 (Lower CI= -1.90, Upper CI=2.74); Number of litters per year effect size: bats =0.05 (Lower CI= -2.38, Upper CI=3.89); primates= -0.09 (Lower CI= -1.08, Upper CI=1.68); See R code provided in supplement for details).


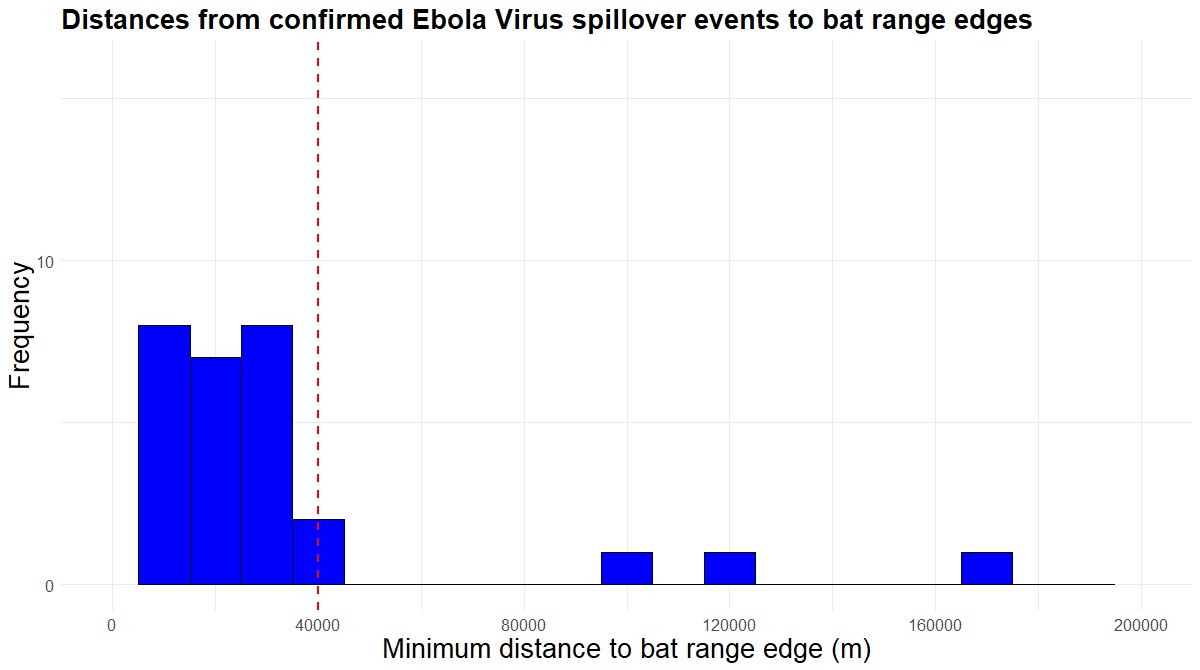


**Fig. S1:** Miminum distance from each confirmed Ebola outbreak from any bat species range-edge.


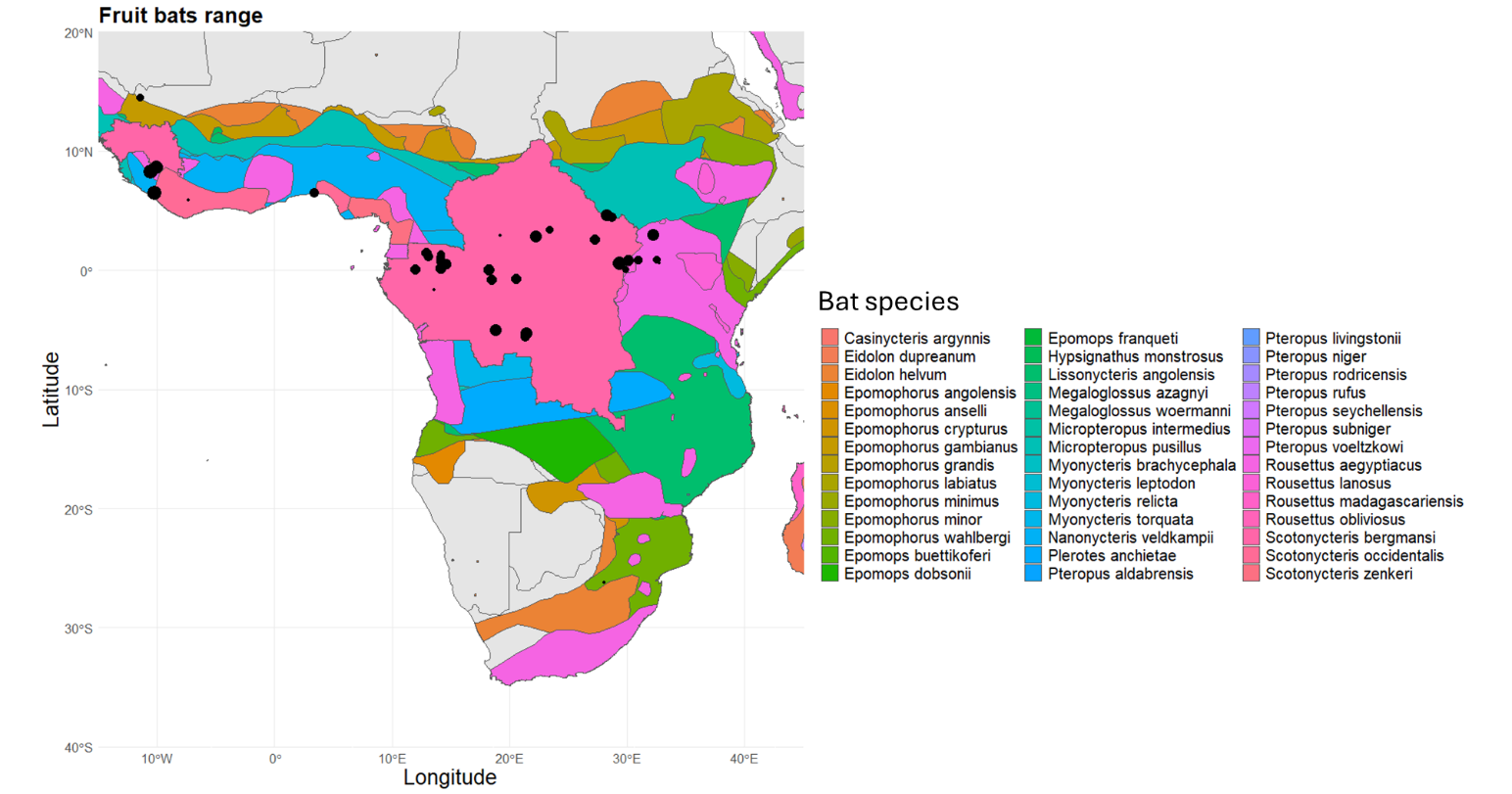


**Figure S2:** *Confirmed Ebola outbreaks with fruit bat species ranges.* Black dots represent confirmed Ebolavirus spillover events, with their size proportional to the number of deaths at each location.


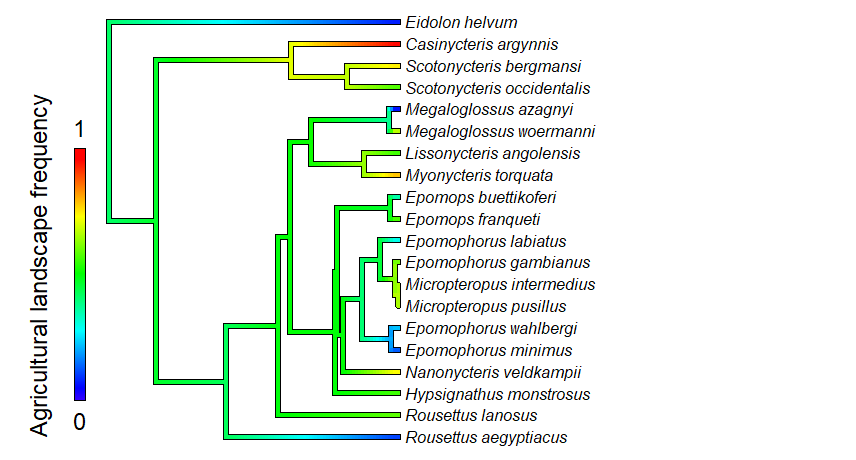


**Figure S3.1:** *Agricultural landscape frequency of all fruit bat species investigated in this study*. Figure created with R package Phytool


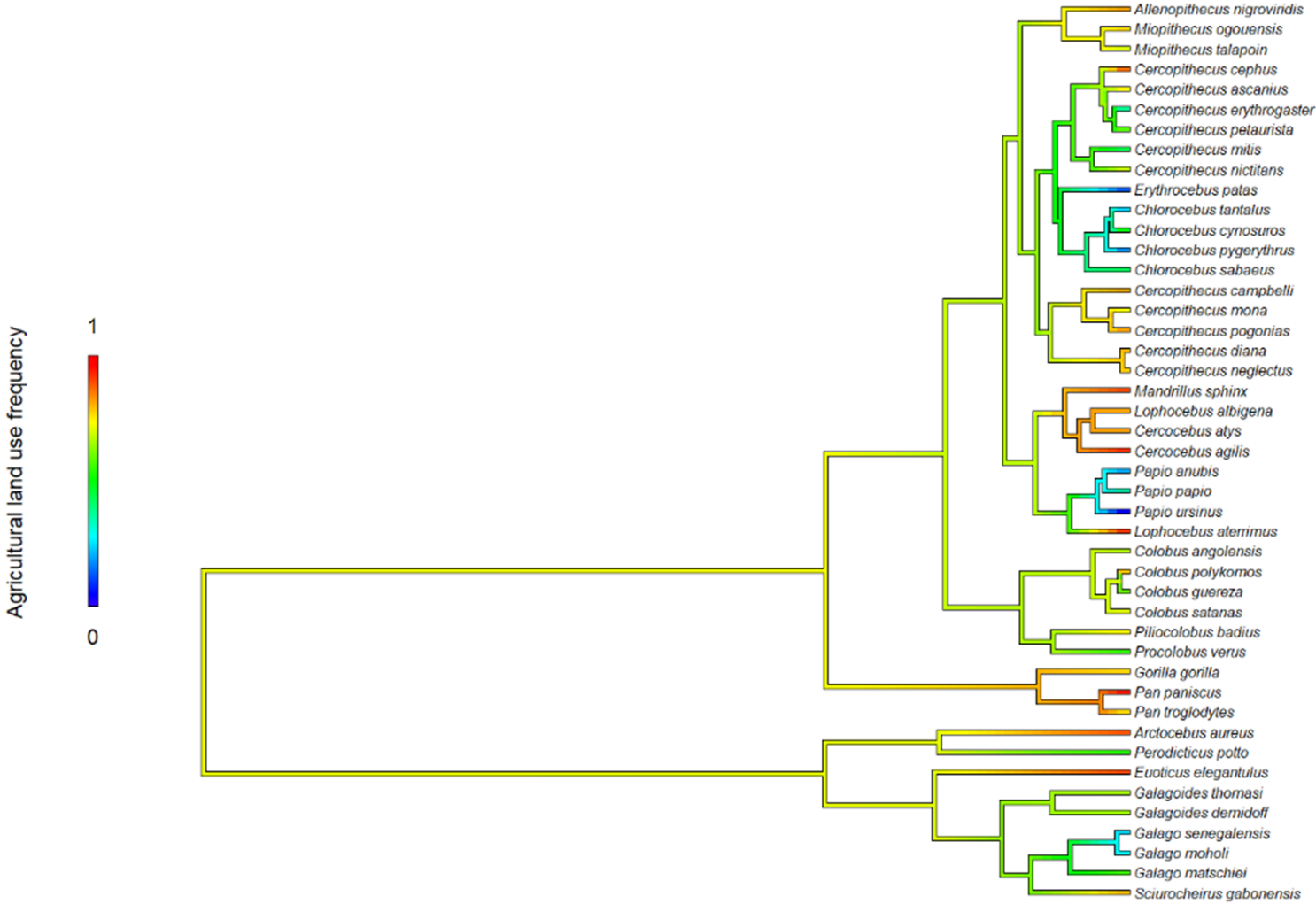


**Figure S3.2**: *Agricultural landscape frequency of all primate species investigated in this study*. Figure created with R package Phytool


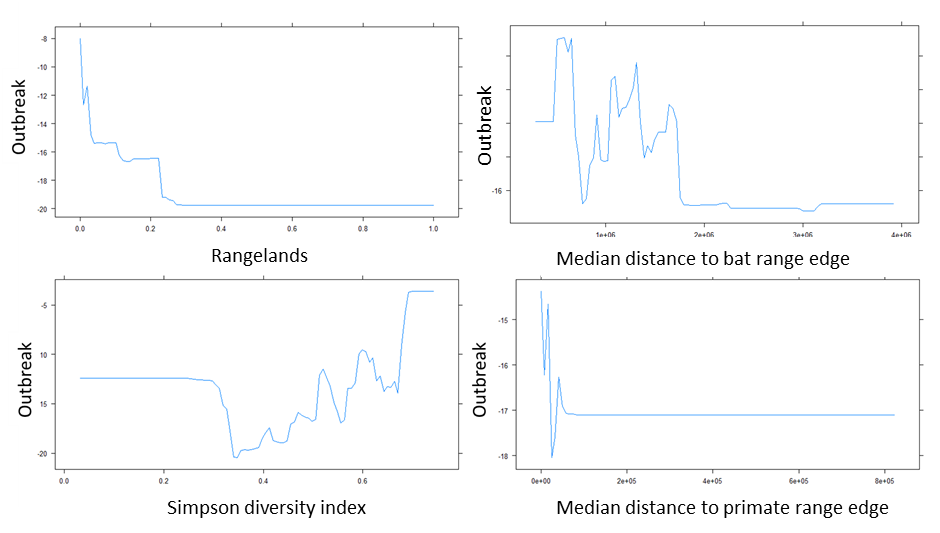


**Fig S4**: *Raw marginal plots from the GBM analysis*. Marginal plots for distances should be interpret in reverse order from the toon graphics provided in Table 1, as proximity to species range edge is on the far left. Note: these marginal plots were extracted using the plot.gbm function (package: GBM), as Caret doesn’t have an implemented method to do so.


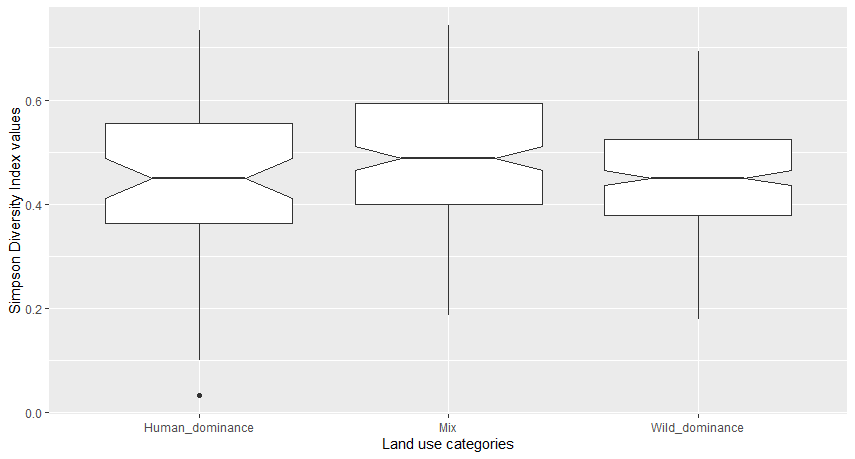


**Figure S5:** *Simpson diversity index for each general land use categories*. Bins go as follow: Human dominance: 80-100% of human associated land cover, Mix: Mix (20-80%) proportion of human and Wild land cover, Wild dominance: 80-100% of wild or low human population density land cover.


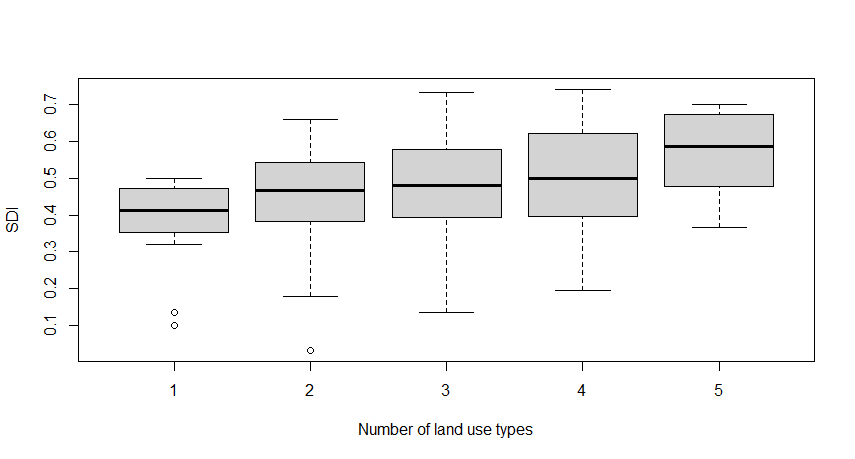


**Figure S6:** Simpson diversity index per number of land use types according to the SEDAC database.


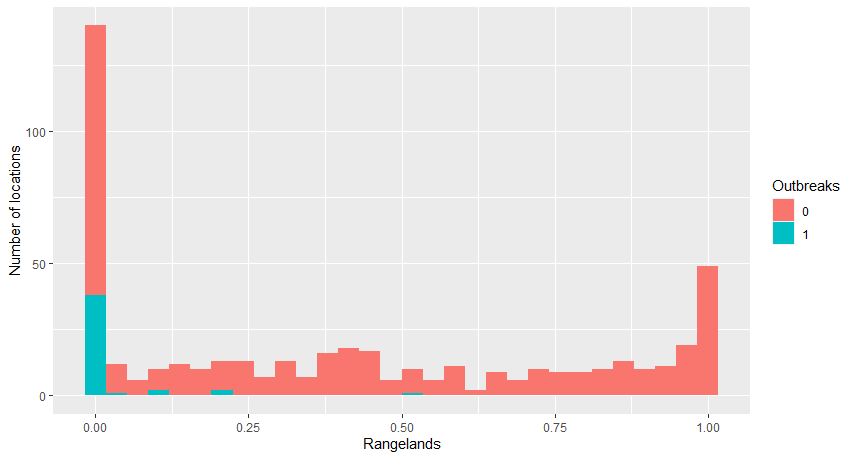


**Figure S7:** Number of locations per proportion of Rangeland in their 40km buffer.

**Table S1**: ANOVA and Post-Hoc Tukey honestly significant difference (HSD) results for Simpson’s diversity index.

| Pairs | Lower interval | Upper interval | p-value |
| --- | --- | --- | --- |
| Mix-Human dominance | 0.002337377 | 0.07960899 | 0.0346 |
| Wild dominance-Human dominance | -0.038858387 | 0.03557628 | 0.9940 |
| Wild dominance-Mix | -0.068615877 | -0.01661259 | 0.0003 |
